# MicroRNA-199a-3p and microRNA-34a regulate apoptosis in human osteosarcoma cells

**DOI:** 10.1042/BSR20140084

**Published:** 2014-08-06

**Authors:** Ye Tian, Ying-Ze Zhang, Wei Chen

**Affiliations:** *Department of Orthopaedic Surgery, the Third Hospital of HeBei Medical University, Shijiazhuang, China

**Keywords:** apoptosis, human osteosarcoma cells, microRNA-199a, microRNA-34a, DMEM, Dulbecco’s modified Eagle’s medium, miRNA, microRNA, mTOR, mammalian target of rapamycin, MTT, 3-(4, 5-dimethylthiazol-2-yl)-2, 5-diphenyltetrazolium bromide, nt, nucleotide, qPCR, quantative PCR, RT, reverse transcription, TUNEL, terminal deoxynucleotidyl transferase-mediated dUTP nick-end labelling, UTR, untranslated region

## Abstract

miRNAs (microRNAs) are small non-coding RNAs [18–25 nt (nucleotides)] that regulate gene expression mainly through affecting post-transcriptional modification. Osteosarcoma is an aggressive sarcoma of the bone characterized by a high level of genetic instability and recurrent DNA deletions and amplifications. miRNAs play an important role in cancer cell growth and migration; however, the potential roles of miRNAs in osteosarcoma remain largely uncharacterized. In this paper, miR-199a and miR-34a were discussed the mechanisms of apoptosis using miRNA mimics in human osteosarcoma cells. The results demonstrated that miR-199a and miR-34a could induce the apoptosis of human osteosarcoma cells via p53 signalling pathway.

## INTRODUCTION

Cancers share a number of characteristics, such as the loss of cellular identity, an increased ability to grow and proliferate and alterations in the systems controlling cell death. Studies performed in a variety of organisms have revealed that miRNAs (microRNAs) have the ability to regulate these cellular processes, suggesting that they could be involved in cancer [1,2]. The miRNAs are small non-coding RNAs of 18–25 nt (nucleotides), typically excised from 60 to 110 nt fold-back RNA precursor structures [3–5]. The biogenesis of miRNAs involves a complex protein system, including members of the Argonaute family, Pol II-dependent transcription and the RNase IIIs Drosha and Dicer [6]. To date, every type of cancers analysed by miRNA profiling has shown significantly different miRNA profiles compared with normal cells from the same tissues. Several other genome-wide profiling studies have been performed on various cancer types, including chronic lymphocytic leukemia [7], breast cancer [8], glioblastoma [9], hepatocellular carcinoma [10], lung cancer [11], colon cancer and endocrine pancreatic tumours [12].

Osteosarcoma is the most common malignant bone tumour in adolescent. Although many tumours initially respond to chemotherapy, patients with metastatic disease and relapsed disease continue to have extremely poor survival outcomes [13]. For patients having no metastatic disease at diagnosis, the 5-year survival is 60–70% [14], for patients who present with metastatic disease or whose tumour recurs, the clinical outcomes are far worse [15]. However, the mechanisms that orchestrate the multiple oncogenic insults required for initiation and progression of osteosarcoma are not clear. MiRNAs play important roles in several cellular processes, such as proliferation, differentiation, apoptosis and development, by simultaneously controlling the expression levels of hundreds of genes [4,16]. MiRNAs have demonstrated far-reaching effects on the cellular biology of development and cancer [17,18]. Their role in osteosarcoma genesis remains largely unexplored. MiR-199a and miR-34a has showed it could repress the cancer cell growth and migration respectively in previous study [19–21]. The p53 tumour suppressor gene encodes a transcription factor that is translationally and post-translationally activated following DNA damage and oncogene activation [22,23]. In the previous study, overexpression of the primary miR-34a transcript was induced after p53 activation and by DNA damage in a p53-dependent manner in cancer cells [22]. In the other report, overexpression of miR-199a-3p leads to inhibition of cell migration and cell growth, increase of G1-phase cell population [21]. Here, the miR-199a and miR-34a were transfected together to human osteosarcoma cells to investigate the mechanisms of suppress proliferation and apoptosis.

## MATERIALS AND METHODS

### Cell culture

Human osteosarcoma cell lines MG63 was obtained from ATCC (American Type Culture Collection) and cultured in H-DMEM (Dulbecco's modified Eagle's medium) (Gibco) supplemented with heat-inactivated 10% FBS (Gibco) at 37°C in a humidified incubator containing 5% (v/v) CO_2_.

### MiRNA mimics synthesis and transfection

MiRNA mimics were synthesized by Thermo Fisher and transfected using Lipofectamine 2000 (Invitrogen). The oligonucleotide sequence of the hsa-miR-199a-3p mimics (MIMAT0000232) and has-miR-34a (MIMAT0000255) were: 5′-ACAGUAGUCUGCACAUUGGUUA-3′ and 5′-UGGCAGUGUCUUAGCUGGUUGU-3′. A scrambled siRNA sequence (5′-TTCTCCGAACGTGTCACGT-3′) was used as the negative control. Cells were cultured in complete medium after transfected 6 h.

### RNA isolation and qPCR (quantative PCR) analysis of miRNA

Total RNA, including miRNAs, was isolated from cell lines using TRIzol reagent (Invitrogen) according to the manufacturer's instructions. cDNA synthesis was carried out with the High Capacity cDNA synthesis kit (Applied Biosystems) using 2 ng of total RNA as template. The miRNA sequence-specific RT (reverse transcription)-PCR primers for miR-199a-3p, miR-34a and endogenous control U6 were purchased from Ambion. Real-time qRT-PCR analysis was carried out using Applied Biosystems 7500 real-time PCR system. The gene expression threshold cycle (C_T_) values of miRNAs from each sample were calculated by normalizing with internal control U6 and relative quantitation values were plotted.

### Cell growth assays

Equal numbers of human osteosarcoma cells were plated in triplicate in 96-well culture plates and stained with MTT [3-(4, 5-dimethylthiazol-2-yl)-2, 5-diphenyltetrazolium bromide] after transfection. Six replicates were prepared for transfected and cultured until 7 days. After the addition of 200 μl DMSO (dymethylsulfoxide) in each well, the samples were incubated in the dark for 30 min, and then swirled for mixing. Absorbance A at 490 nm was measured using enzymatic reader. Experiments were repeated three times.

### TUNEL (terminal deoxynucleotidyl transferase-mediated dUTP nick-end labelling) analysis

The apoptosis of human osteosarcoma cells were detected using TdT (terminal deoxynucleotidyl transferase)-mediated TUNEL technique after miRNAs transfection of 72 h. Briefly, the human osteosarcoma cells were fixed for 1 h at room temperature and then incubated in permeabilization solution for 2 min on ice. The subsequent staining was carried out according to the manufacturer's instructions.

### Western blot analysis

Cells were lysed using M-PER Protein Extraction Reagent (Pierce) supplemented with protease inhibitor cocktail (DMSF). Protein concentrations of the extracts were measured with BCA assay (Pierce) and equalized with the extraction reagent. Equal amount of the extracts were loaded and subjected to SDS/PAGE, transferred onto nitrocellulose membranes, and then blotted as previously reported [24]. Specific antibodies and horseradish peroxidase-coupled secondary antibodies were purchased from Santa Cruz. Membranes were probed using ultra-enhanced chemiluminescence Western blotting detection reagents. GAPDH (glyceraldehyde-3-phosphate dehydrogenase) was used as internal control.

### Real-time PCR

Real-time PCR was carried out using a DyNAmo SYBR Green qPCR kit (Finnzymes). The PCR mixture, which contained 20 pmol of forward and reverse primers and 2 μl of cDNA, was subjected to amplification with a DNA Engine Opticon 1 (MJ Research). The cycles were set at 95°C for 10 min for preheating, followed by 40 cycles at 94°C for 15 s, at 55°C for 30 s and at 72°C for 30 s. The amplicons were detected directly by measuring the increase in fluorescence caused by the binding of the SYBR Green I dye to gene-specific and amplified double-strand DNA using a DNA Engine Opticon 1. Following the completion of the PCR reaction, the temperature was raised from the annealing temperature to 95°C for melting curve analysis. The expression level was calculated by the 2^−ΔΔCt^ method and compared with the relative expression. Primer sequences are shown in Table 1.

**Table 1:** Primer sequences used in RT–PCR and qPCR assay

Gene	Forward/reverse	Primer sequence	T_m_ (°C)	Cycle	Product size (bp)
Stat 3	Forward	5′-GAAAGCCTGCCGGTGACTAA-3′	60	30	115
	Reverse	5′-GCCCAATACGACCAAATCAGAGA-3′			
p53	Forward	5′-CCAGTCACAGCAGCACAGAT-3′	60	30	215
	Reverse	5′-ACCGTCTCGGTTTTCACTGC-3′			
Bcl-2	Forward	5′-GAATGGGCAGCCGTTAGGAA-3′	60	30	168
	Reverse	5′-CCCAATACGACCAAATCAGAGA-3′			
Bax	Forward	5′-AGGGTGTAAAACGCAGCTCA-3′	60	30	202
	Reverse	5′-AGGGTGTAAAACGCAGCTCA-3′			
Bcl-xl	Forward	5′-AGGGACTGCACAGTCAATGG-3′	60	30	162
	Reverse	5′-CCATGTTCACATCATGTCCTTCA-3′			
MCL-1	Forward	5′-TCCTTCCTGGGTATGGAATCCT-3′	60	30	113
	Reverse	5′-GCTCAGTAACAGTCCGCCTA-3′			
GAPDH	Forward	5′-CCCGTTGCTGTCGCCCGTTC-3′	60	30	149
	Reverse	5′-GCCTTGACCGTGCCGTGGAA-3′			

## RESULTS

### MiR-199a-3p and miR-34a expression after transfection in human osteosarcoma cells

**Figure 1:**
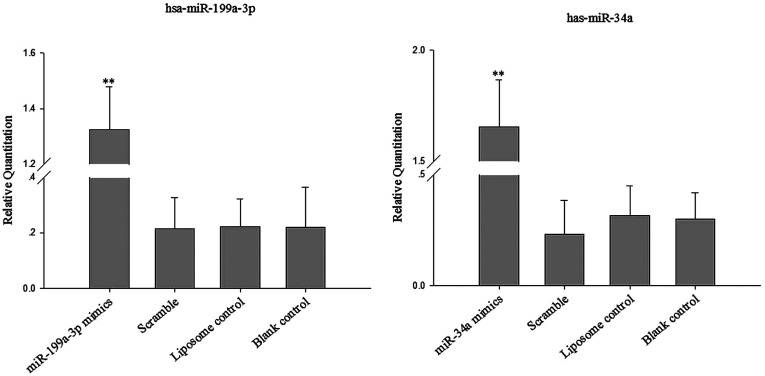
MiR-199a-3p and miR-34a mRNA up-regulation in miR-199a-3p and miR-34a mimics transfected human osteosarcoma cells Cells were incubated with synthetic oligonucleotides as described in the Materials and Methods section and miR-199a-3p and miR-34a mRNA was quantified by real-time PCR. Data represent mean±S.D. of four independent experiments miR-199a-3p and miR-34a mRNA was increased in miR-199a-3p and miR-34a mimics-treated cells compared with the blank control, liposome control and scramble groups (*P*<0.01)

To determine the functional role of miR-199a-3p and miR-34 in human osteosarcoma cells, we stably transfected miR-199a-3p and miR-34a mimics into human osteosarcoma cells. The expression of miR-199a-3p and miR-34a was quantified by qRT-PCR 72 h after transfection. As shown in Figure 1, miR-199a-3p and miR-34a levels were significantly elevated by the miRNA mimics.

### MiR-199a-3p and miR-34a inhibited the proliferation of human osteosarcoma cells *in vitro*

**Figure 2:**
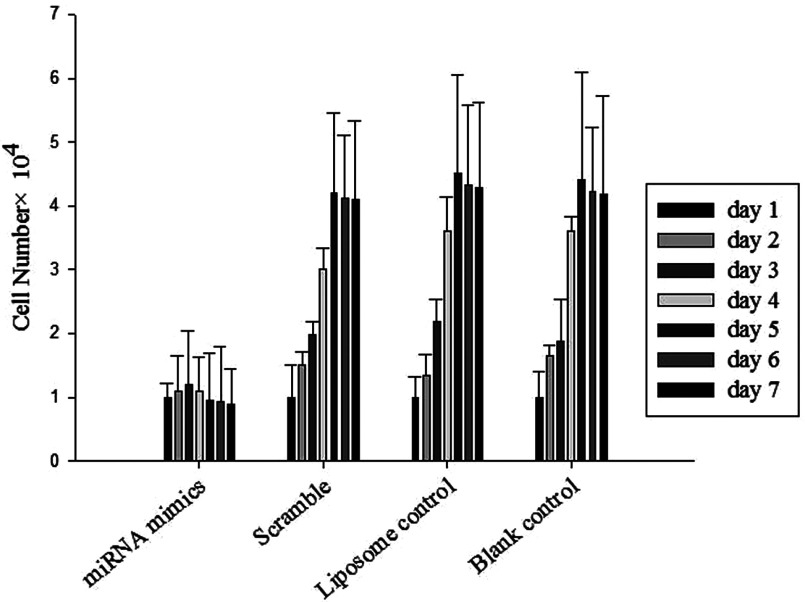
Human osteosarcoma cells were transfected with miR-199a-3p and miR-34a mimics and cell proliferation was assessed using the MTT assay Data are presented as the mean of triplicate experiments. The growth inhibitory effect of the miR-199a-3p and miR-34a mimics was time-dependent, with the maximum inhibition detected 7 days after transfection. Significant difference (*P*<0.01)

The MTT assay was used to measure the cell growth of human osteosarcoma cells 3 days after transfection with miR-199a-3p and miR-34a mimics oligonucleotides in comparison with black control and scrambled control. As shown in Figure 2, the growth inhibitory effect of the miR-199a-3p and miR-34a mimics was time-dependent, with the maximum inhibition detected 7 days after transfection. These results suggest that miR-199a-3p and miR-34a might function as a novel tumour suppressor in human osteosarcoma.

### MiR-199a-3p and miR-34a target genes expression in human osteosarcoma cells

**Figure 3:**
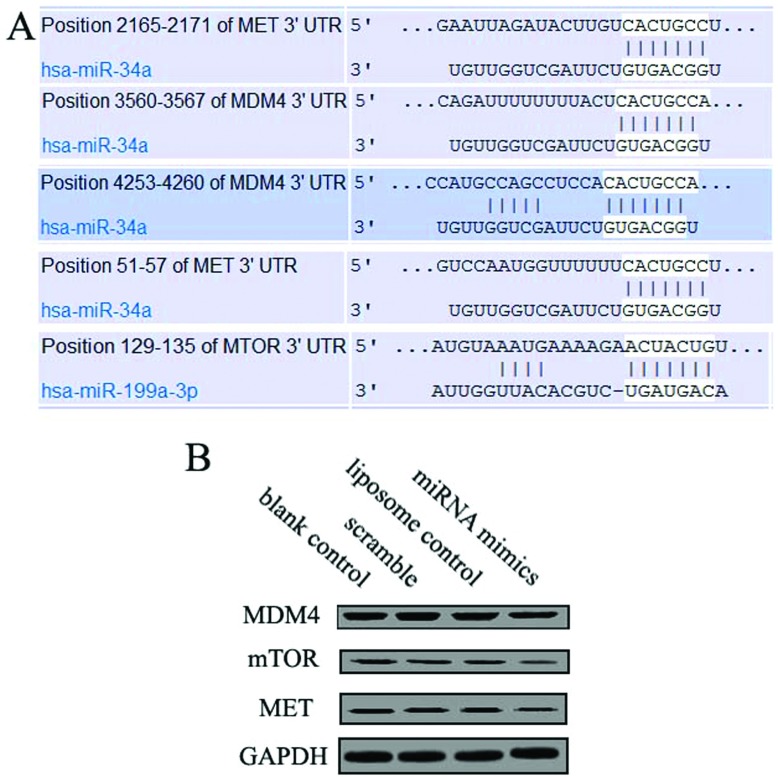
Effect of miR-199a-3p and miR-34a mimics on MDM4, MET and mTOR expression Human osteosarcoma cells were treated with synthetic oligonucleotides as described in the Materials and Methods section. miR-199a-3p and miR-34a mimics down-regulated MDM4, MET and mTOR expression compared with the blank control, scrambled, liposome control and oligonucleotides. (**A**) The position of target site was combined by miRNAs. (**B**) Expression level of target protein was detected by Western blot.

We then analysed the human miR-199a-3p and miR-34a sequence and its target gene sequence, confirmed that mTOR (mammalian target of rapamycin), MET and MDM4 were target genes for that miRNA s. Figure 3 showed that miR-199a-3p and miR-34a targeted mTOR, MET and MDM4 at the 3′-UTR (untranslated region) in human osteosarcoma cells to regulate translation. Protein expression of mTOR, MET and MDM4, putative target genes was performed on miR-199a-3p and miR-34a transfected cells according to Gel-Pro Analyzer 4 comparative method.

### Detection of apoptosis and p53 pathway

**Figure 4:**
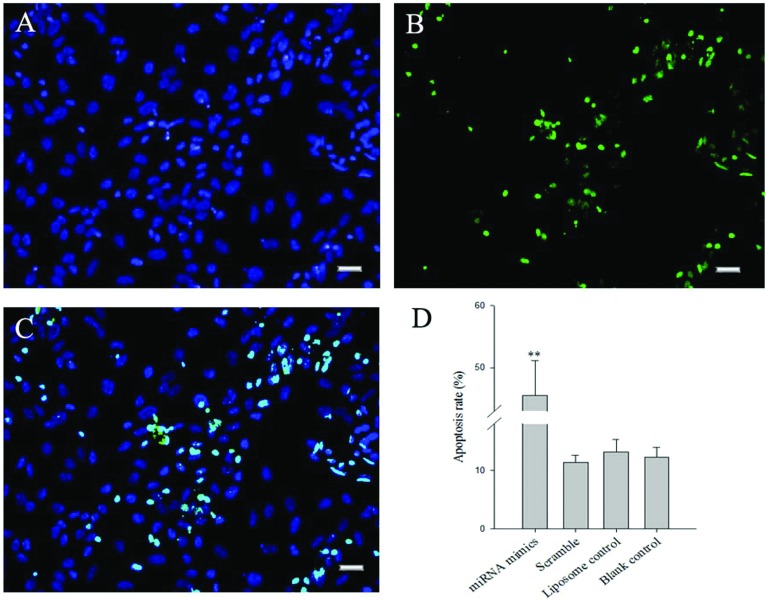
Effect of miRNAs mimics on cell apoptosis of human osteosarcoma cells after transfected using the TUNEL method TUNEL is a common method for detecting DNA fragmentation that results from apoptotic signalling cascades. Positive cells were stained into green, indicating that DNA fragmentation had taken place. This symbolic apoptotic event was greatly reduced with miR-199a-3p and miR-34a. Bar=50 μm

Human osteosarcoma cells were transfected after 7 days and analysed by TUNEL. TUNEL is a common method for detecting DNA fragmentation that results from apoptotic signalling cascades. DNA fragmentation was labelled *in situ* via TUNEL assay (Figure 4). It could be observed that there were obviously increased positive cells in the treated groups, and their proportion, to the contrary of the strongly positive result of the contrast group.

**Figure 5:**
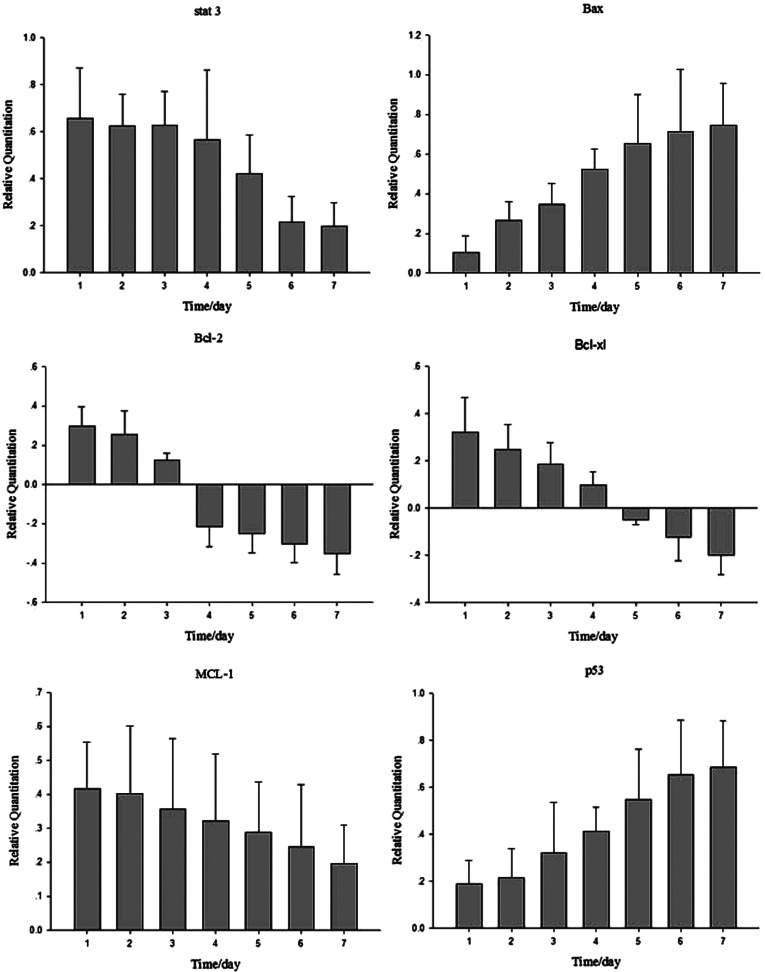
Real-time PCR analyses expression of p53 pathway members The p53 pathway members expression of human osteosarcoma cells at 7 days after transfection. Stat 3, Bcl-2, Bcl-xl and MCL-1 were detected, and the gene expression level showed a time-lapse decrease; however, the gene expression of Bax and p53 showed a time-lapse increase.

The expressions of members of apoptosis pathway were analysed in human osteosarcoma cells treated with miR-199a-3p and miR-34a mimics or scrambled oligonucleotides using real-time PCR. Transfection with miR-199a-3p and miR-34a mimics oligonucleotides as a result down-regulated mTOR (regulon of cell proliferation), MET (regulon of cell proliferation) and MDM4 (repressor of p53) expression to activate the p53-apoptosis pathway. These results suggested that the osteosarcoma suppressor activity of miR-199a-3p and miR-34a in human osteosarcoma cells may be associated with p53 pathway. Transfection of miR-199a-3p and miR-34a mimics oligonucleotides into human osteosarcoma cells significantly decreased cell growth and increased cell apoptosis, thus indicating that the inhibition effect is associated with an activation of p53-apoptosis pathway (Figure 5).

## DISCUSSION

MiRNAs are small non-coding RNA that exhibit a high degree conservation of structure and function in plant and animal. They exist in two forms of pre-miRNAs and mature miRNAs, and only the mature miRNAs mediated by the two RNase III endonucleases Dicer and Drosha [4], play a key biological role. The mature miRNAs inhibit protein translation through binding the 3′-UTR of target mRNA partly, while they induce target mRNA cleavage through binding mRNA with perfect complementarity [25,26]. A target gene analysis of miRNAs may lead to better understanding of the mechanisms by which miRNAs mediate proliferation and apoptosis in cancer cells. It was found to down-regulate the oncogenes after the target gene analysis of miR-199a-3p and miR-34a using TargetScan tools, such as Met, mTOR and MDM4. The proto-oncogene MET is the hepatocyte growth factor receptor and encodes tyrosine-kinase activity. The primary single-chain precursor protein is post-translationally cleaved to produce the alpha and beta subunits, which are disulphide linked to form the mature receptor. Various mutations in the MET gene are associated with carcinoma occurrence. The mTOR is a serine/threonine protein kinase that regulates cell growth, cell proliferation, cell motility, cell survival, protein synthesis and transcription [31].

The miR-199a-3p decreases the expression of several oncogenes and anti-apoptotic genes, including Met and mTOR. These results are consistent with several studies that have suggested that miR-199a-3p is a potential tumour suppressor [27–29]. First, the expression of miR-199a-3p is decreased in all proliferating cell lines tested except for fibroblasts. Secondly, introduction of the miR-199a-3p precursor induced apoptosis in cancer cells. Thirdly, miR-199a-3p down-regulates both Met proto-oncogenes [21,30]. In previously, many reports surfaced that the miR-34 family are direct p53 targets, which induce cell-cycle arrest, senescence and apoptosis [31–33]. In animals, miR-34 diverged into a family of three homologous miRNAs: MiR-34a, MiR-34b and MiR-34c. The mature MiR-34a sequence is located within the second exon of its non-coding host gene, which contains a predicted p53 binding site [22,31–33]. Many groups have reported the association of miR-34 with tumourigenesis. Decreased expression of miR-34 was found in neuroblastoma, non-small-cell lung cancer and pancreatic cancer [31,32]. Minimal deletions and epigenetic inactivations of miR-34 were found in breast, colon and lung cancers as well as melanoma [34]. Our research demonstrated that miR-199a-3p and miR-34a could down-regulate its target genes, mTOR, MET and MDM4 in human osteosarcoma cells. mTOR and MET had important roles in cell cycle, the knockout of mTOR and MET could induce the cell-cycle arrest and senescence. MDM4 was an inhibitor factor of *p53* gene, up-regulation of *p53* could induce cell apoptosis. So, our studies suggest that miR-199a-3p and miR-34a may play an important role in the pathogenesis of cancers, especially osteosarcoma.

In conclusion, in this study, we provide direct evidence that miR-199a-3p and miR-34a influences cell apoptosis. The function of miR-199a-3p and miR-34a in cell proliferation and apoptosis, it was clearly dependent on the presence of mTOR, MET and MDM4 gene in human osteosarcoma cells. Future studies are needed to further define the additional molecular pathways mediated by miR-199a-3p and miR-34a.

## References

[1] Stahlhut Espinosa C. E., Slack F. J. (2006). The role of microRNAs in cancer. Yale J. Biol. Med..

[2] Esquela-Kerscher A., Slack F. J. (2006). Oncomirs–microRNAs with a role in cancer. Nat. Rev. Cancer.

[3] Ambros V. (2004). The functions of animal microRNAs. Nature.

[4] Bartel D. P. (2004). MicroRNAs: genomics, biogenesis, mechanism, and function. Cell.

[5] Pasquinelli A. E., Hunter S., Bracht J. (2005). MicroRNAs: a developing story. Curr. Opin. Genet. Dev..

[6] Kim V. N., Nam J. W. (2006). Genomics of microRNA. Trends Genet..

[7] Calin G. A., Liu C. G., Sevignani C., Ferracin M., Felli N., Dumitru C. D., Shimizu M., Cimmino A., Zupo S., Dono M. (2004). MicroRNA profiling reveals distinct signatures in B cell chronic lymphocytic leukemias. Proc. Natl. Acad. Sci. U.S.A..

[8] Iorio M. V., Ferracin M., Liu C. G., Veronese A., Spizzo R., Sabbioni S., Magri E., Pedriali M., Fabbri M., Campiglio M. (2005). MicroRNA gene expression deregulation in human breast cancer. Cancer Res..

[9] Ciafre S. A., Galardi S., Mangiola A., Ferracin M., Liu C. G., Sabatino G., Negrini M., Maira G., Croce C. M., Farace M. G. (2005). Extensive modulation of a set of microRNAs in primary glioblastoma. Biochem. Biophys. Res. Commun..

[10] Murakami Y., Yasuda T., Saigo K., Urashima T., Toyoda H., Okanoue T., Shimotohno K. (2006). Comprehensive analysis of microRNA expression patterns in hepatocellular carcinoma and non-tumorous tissues. Oncogene.

[11] Yu S. L., Chen H. Y., Chang G. C., Chen C. Y., Chen H. W., Singh S., Cheng C. L., Yu C. J., Lee Y. C., Chen H. S. (2008). MicroRNA signature predicts survival and relapse in lung cancer. Cancer Cell.

[12] Roldo C., Missiaglia E., Hagan J. P., Falconi M., Capelli P., Bersani S., Calin G. A., Volinia S., Liu C. G., Scarpa A., Croce C. M. (2006). MicroRNA expression abnormalities in pancreatic endocrine and acinar tumors are associated with distinctive pathologic features and clinical behavior. J. Clin. Oncol..

[13] Kansara M., Thomas D. M. (2007). Molecular pathogenesis of osteosarcoma. DNA Cell Biol..

[14] Ferrari S., Palmerini E., Staals E. L., Mercuri M., Franco B., Picci P., Bacci G. (2009). The treatment of nonmetastatic high grade osteosarcoma of the extremity: review of the Italian Rizzoli experience. Impact on the future. Cancer Treat. Res..

[15] Ferguson W. S., Goorin A. M. (2001). Current treatment of osteosarcoma. Cancer Invest..

[16] He L., Hannon G. J. (2004). MicroRNAs: small RNAs with a big role in gene regulation. Nat. Rev. Genet..

[17] Jones K. B., Salah Z., Del Mare S., Galasso M., Gaudio E., Nuovo G. J., Lovat F., LeBlanc K., Palatini J., Randall R. L. (2012). miRNA signatures associate with pathogenesis and progression of osteosarcoma. Cancer Res..

[18] Maire G., Martin J. W., Yoshimoto M., Chilton-MacNeill S., Zielenska M., Squire J. A. (2011). Analysis of miRNA-gene expression-genomic profiles reveals complex mechanisms of microRNA deregulation in osteosarcoma. Cancer Genet..

[19] Raver-Shapira N., Marciano E., Meiri E., Spector Y., Rosenfeld N., Moskovits N., Bentwich Z., Oren M. (2007). Transcriptional activation of miR-34a contributes to p53-mediated apoptosis. Mol. Cell.

[20] He C., Xiong J., Xu X., Lu W., Liu L., Xiao D., Wang D. (2009). Functional elucidation of MiR-34 in osteosarcoma cells and primary tumor samples. Biochem. Biophys. Res. Commun..

[21] Duan Z., Choy E., Harmon D., Liu X., Susa M., Mankin H., Hornicek F. (2011). MicroRNA-199a-3p is downregulated in human osteosarcoma and regulates cell proliferation and migration. Mol. Cancer Ther..

[22] Tarasov V., Jung P., Verdoodt B., Lodygin D., Epanchintsev A., Menssen A., Meister G., Hermeking H. (2007). Differential regulation of microRNAs by p53 revealed by massively parallel sequencing: miR-34a is a p53 target that induces apoptosis and G1-arrest. Cell Cycle.

[23] Vogelstein B., Lane D., Levine A. J. (2000). Surfing the p53 network. Nature.

[24] Hou J., Wang P., Lin L., Liu X., Ma F., An H., Wang Z., Cao X. (2009). MicroRNA-146a feedback inhibits RIG-I-dependent Type I IFN production in macrophages by targeting TRAF6, IRAK1, and IRAK2. J. Immunol..

[25] Pillai R. S. (2005). MicroRNA function: multiple mechanisms for a tiny RNA?. RNA.

[26] Zamore P. D., Haley B. (2005). Ribo-gnome: the big world of small RNAs. Science.

[27] Ichimi T., Enokida H., Okuno Y., Kunimoto R., Chiyomaru T., Kawamoto K., Kawahara K., Toki K., Kawakami K., Nishiyama K. (2009). Identification of novel microRNA targets based on microRNA signatures in bladder cancer. Int. J. Cancer.

[28] Kim S., Lee U. J., Kim M. N., Lee E. J., Kim J. Y., Lee M. Y., Choung S., Kim Y. J., Choi Y. C. (2008). MicroRNA miR-199a* regulates the MET proto-oncogene and the downstream extracellular signal-regulated kinase 2 (ERK2). J. Biol. Chem..

[29] Fornari F., Milazzo M., Chieco P., Negrini M., Calin G. A., Grazi G. L., Pollutri D., Croce C. M., Bolondi L., Gramantieri L. (2010). MiR-199a-3p regulates mTOR and c-Met to influence the doxorubicin sensitivity of human hepatocarcinoma cells. Cancer Res..

[30] Lee Y. B., Bantounas I., Lee D. Y., Phylactou L., Caldwell M. A., Uney J. B. (2009). Twist-1 regulates the miR-199a/214 cluster during development. Nucleic Acids Res..

[31] Bommer G. T., Gerin I., Feng Y., Kaczorowski A. J., Kuick R., Love R. E., Zhai Y., Giordano T. J., Qin Z. S., Moore B. B. (2007). p53-mediated activation of miRNA34 candidate tumor-suppressor genes. Curr. Biol..

[32] Chang T. C., Wentzel E. A., Kent O. A., Ramachandran K., Mullendore M., Lee K. H., Feldmann G., Yamakuchi M., Ferlito M., Lowenstein C. J. (2007). Transactivation of miR-34a by p53 broadly influences gene expression and promotes apoptosis. Mol. Cell.

[33] Corney D. C., Flesken-Nikitin A., Godwin A. K., Wang W., Nikitin A. Y. (2007). MicroRNA-34b and MicroRNA-34c are targets of p53 and cooperate in control of cell proliferation and adhesion-independent growth. Cancer Res..

[34] Calin G. A., Sevignani C., Dumitru C. D., Hyslop T., Noch E., Yendamuri S., Shimizu M., Rattan S., Bullrich F., Negrini M., Croce C. M. (2004). Human microRNA genes are frequently located at fragile sites and genomic regions involved in cancers. Proc. Natl. Acad. Sci. U.S.A..

